# NDVI as a potential tool for forecasting changes in geographical range of sycamore (*Acer pseudoplatanus* L.)

**DOI:** 10.1038/s41598-023-46301-x

**Published:** 2023-11-14

**Authors:** Monika Konatowska, Adam Młynarczyk, Wojciech Kowalewski, Paweł Rutkowski

**Affiliations:** 1 Department of Botany and Forest Habitats, Faculty of Forestry and Wood Technology, Poznań University of Life Sciences, Wojska Polskiego 71F, 60-625 Poznan, Poland; 2 Environmental Remote Sensing and Soil Science Research Unit, Faculty of Geographic and Geological Sciences, Adam Mickiewicz University in Poznań, Wieniawskiego 1, 61-712 Poznan, Poland; 3 Department of Artificial Intelligence, Faculty of Mathematics and Computer Science, Adam Mickiewicz University in Poznań, Wieniawskiego 1, 61-712 Poznan, Poland

**Keywords:** Climate-change ecology, Forest ecology, Forestry

## Abstract

Determining the natural range of *Acer pseudoplatanus* and the future directions of its spread is not clear. Modern technological achievements, including tools related to remote sensing, provide new opportunities to assess the degree of spread and adaptation of species to a changing climate. The aim of the work was to demonstrate the possibility of using NDVI to assess the habitat conditions of sycamore in Poland and the possibility of its natural expansion. The data analysis was divided into 2 parts. The first covered the characteristics of all sycamore stands occurring in Poland. In the second part, the analysis of sycamore stands using NDVI was made. The results of the study show that the highest average NDVI values are found in sycamore stands in the northern part of Poland, which has so far been considered less favorable for sycamore. This may suggest the potential for an increase in the share of sycamore towards the north. The results also confirm the forecasts given in the literature regarding the spread of sycamore towards Lithuania, Latvia and Estonia. The results also point to Denmark and the western part of the British Isles as potentially favorable habitats for sycamore.

## Introduction

Progressing climate change affects, among others, plant ranges^1,2^ and animal migrations^3,4^). One of the important native tree species in the flora of Europe is sycamore (*Acer pseudoplatanus* L.). According to Pasta et al.^5^, the natural distribution range of sycamore includes Central and Eastern Europe, the mountains of Southern Europe, the Caucasus, and northern Minor Asia. Thanks to its economic importance, as well as the natural function it fulfills in the environment, it has spread beyond its natural range, e.g. to United Kingdom and Scandinavia, and to others continents, including North and South America, Australia and Asia. Currently, outside of its natural range, it is often considered an invasive species^6–8^. However, determining the natural range of sycamore and the future directions of its spread is not clear. Tillisch^9^ suggests that it could have been a native species in Denmark, but due to human activity it has been exterminated in a large part of the country. Maps provided by Tillisch, dating from before 1965, also suggest that the range of sycamore’s occurrence may have included the southern part of Great Britain. In effect range of *Acer pseudoplatanus* is controversial and climate changes add further uncertainty to decision of management of sycamore stands. Therefore, it is considered necessary to determine the natural conditions of sycamore occurrence and the impact of climate change on its spread. Modern technological achievements, including tools related to remote sensing, also provide new opportunities to assess the degree of spread and adaptation of species to a changing climate. The aim of the work was to demonstrate the possibility of using NDVI to assess the habitat conditions of sycamore in Poland and the possibility of its natural expansion.

The normalized difference vegetation index (NDVI) is commonly used in research, for which the number of publications counts 17.775^10^. NDVI uses land-cover mapping^11–13^, wildfire monitoring^14^, and others. All these papers show that the relationship between NDVI patterns and the state of vegetation is very complex and depends on many factors, including meteorological factors, soil moisture, and type of vegetation cover^13,15–17^, but basically there are no papers related to NDVI and sycamore. Therefore, taking up this topic is considered necessary, especially that the research methods and results can also be applied to other forest trees, not only in Europe.

Many studies^16,18,19^ show that NDVI depends on climatic factors (mainly precipitation and temperature). It can therefore be assumed that changes in NDVI of individual plant species in a given area can be used to monitor the impact of climate change on various plant species or their communities. However, the spread of species is determined not only by climatic conditions, but also by other limiting factors, including forest policy or the impact of herbivores. Therefore, in order to manage the forest environment, it is necessary to take into account all these factors. Undoubtedly, however, the climatic factor is the overriding factor limiting the occurrence of the species in a given area. Therefore, the assessment of the possibility of changing the geographic range of plants is inextricably linked to the prediction of climate change. For sycamore, these forecasts are shown by Mauri et al.^20^). The forecast shows that sycamore should expand its range northwards, gradually covering Lithuania, Latvia, Estonia, and the Scandinavian countries. A significant change is also observed in north-eastern Poland, where, according to the Mauri et al. the conditions are unsuitable for this species. Therefore, the area of Poland was considered representative for assessing the possibility of using NDVI as a tool for forecasting the spread of sycamore at the border of its natural north-eastern range.

**Figure 1:**
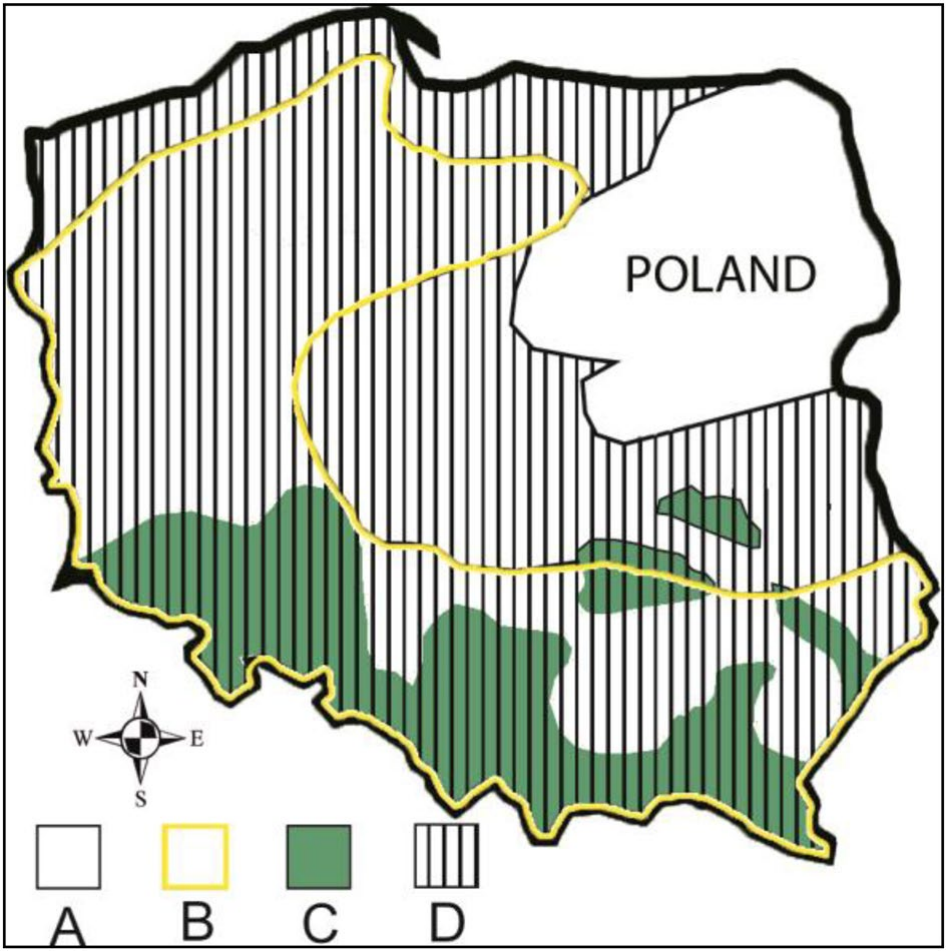
Range of sycamore in Poland according to Szafer^23^ and Boratyński^24^; A—area out of range; B—range according to Szafer^23^; C and D—range according to Boratyński^24^ (C—frequent occurrence; D—diffuse occurrence).

According to Boratyński^21,22^, the boundary of the natural occurrence of sycamore in Poland has been very much blurred as a result of frequent and ancient cultivation and easy wilderness and encroachment into natural forest communities. Therefore, the data on sycamore's range given in the Polish literature may, on the one hand, prove its expansion towards the north-east (Fig. 1), but may also result from differences in the approach to determining the naturalness of its sites.

However, regardless of the views on the natural range of sycamore in Poland and other European countries, it can be considered undisputed that the occurrence of the species in a given region is determined by favorable habitat conditions, of which climatic conditions seem to be the key. Therefore, research on climate change and its impact on the dispersal of species is recognized as the basis for managing the forest environment.

## Methods

All methods were carried out in accordance with relevant guidelines.

The study did not require the collection of plant material, and all data on sycamore were obtained from a database and analyzed on the basis of satellite data.

The data analysis was divided into 2 parts. The first covered the characteristics of all sycamore stands occurring in Poland. It was prepared using traditional methods of inventory of forest areas. In the second part, the analysis of sycamore stands occurring in Poland using NDVI was made, and then, based on data from Poland, the potential of forest habitats was estimated in terms of the possibility of sycamore occurrence throughout Europe.

All tree stands in Poland belonging to the State Forests (over 7 million ha, i.e. 75% of the total forest area in Poland) are subject to a detailed inventory conducted every 10 years. All information obtained during the stocktaking goes to a common database called Forest Data Bank (FDB)^25^ which is at the disposal of the Directorate General of State Forests—the unit managing forests in Poland.

The database used in the study was obtained from the FDB according to 2022. From the database, stands were selected in which the share of sycamore accounted for at least 80% of the share in the upper layer of the stand (a total of 291 units). The criterion of at least 80% was adopted in terms of NDVI used in the work so as to minimize the impact of admixtures of other species on the test results.

The analysis based on data from the forest inventory covered:

1. the share of sycamore in forest habitat types, which are the result of habitat conditions (soil, location, climate), supplemented with a description of vegetation, influencing the selection of species composition as well as the growth and development of trees. Forest habitat types were divided into 3 categories related to geographical location (mountain, upland, and lowland habitats), 5 moisture categories (alluvial, swampy, wet, mesic, dry) and 4 trophic groups (eutrophic, mesotrophic, oligotrophic, poor);
2. age of sycamore stands.

In addition, NDVI was calculated for stands of all main species and compared with the position of sycamore in the species ranking and the distribution of sycamore against the distribution of NDVI in Poland. The decision to take NDVI as an indicator helpful in assessing the moisture status of forest habitats was adopted on the basis of the results of research published by Młynarczyk et al.^13^. Image data from the Sentinel-2 (A, B) satellites of the European Space Agency (ESA) were used for the calculations. Image data were downloaded from Google Earth Engine (GEE) and calculated. The calculations were made using the zonal statistics plug-in, where the boundary layer and the NDVI map were selected in accordance with the methodology from the article by Młynarczyk et al.^13^.

Data on forest stands in Europe, which was used for the analysis of Fig. 5, was taken from Corine Land Cover from 2018^26^ for tree ranges in Europe. On their basis, the calculated NDVI image was cropped to areas covered with trees. A 25 × 25 km grid was created in which the median of data from the excised NDVI layer was placed, which allowed visualization of the data in comparison with the occurrence of sycamore in Europe^27^ and forecasting the potential of sycamore habitats in Poland and Europe.

In order to analyze the collected data, a validation test was conducted using five algorithmic approaches:

1. Linear discriminant analysis (LDA)
2. Quantitative descriptive analysis (QDA)
3. Support vector classifier (SVC)
4. Random Forest
5. Multilayer Perceptron (MLP)

All calculations were performed using Python and the scikit-learn library (cf. https://scikit-learn.org/stable/). The dataset used in the calculations consists of 291 cases, tested in four variants concerning the relation between forest habitat types, the average NDVI value calculated from August 1 to August 14, 2022, the total precipitation from January 1 to August 14, 2022, the average air temperature from January 1 to August 14, 2022, and the average soil moisture from August 1 to August 14, 2022.

The data was split into a training set and a validation set in an 8:2 ratio. The soil moisture, temperature, and precipitation data were retrieved from Google Earth Engine using the dataset “ECMWF/ERA5_LAND/HOURLY.” “ERA5-Land is a reanalysis dataset providing a consistent view of the evolution of land variables over several decades at an enhanced resolution compared to ERA5”^28^. Soil moisture was retrieved at 3 levels (volumetric_soil_water_layer): 0–7 cm, 7–28 cm, 28–100 cm. Temperature (temperature_2m) was averaged, and precipitation (total_precipitation) was summed.

The period for which the data was retrieved was chosen based on the assumption that NDVI during the summer period reflects the condition of forest stands, influenced not only by current weather conditions but also by the state of habitat moisture related to water accumulation in the soil from winter to summer.

The downloaded data was imported into QGIS 3.28. Using the zonal statistics and raster layers tools, the data was clipped based on vector data obtained from the Forest Data Bank. The entire dataset was saved in CSV format for further statistical computations.

### Used software

**Figure 2:**
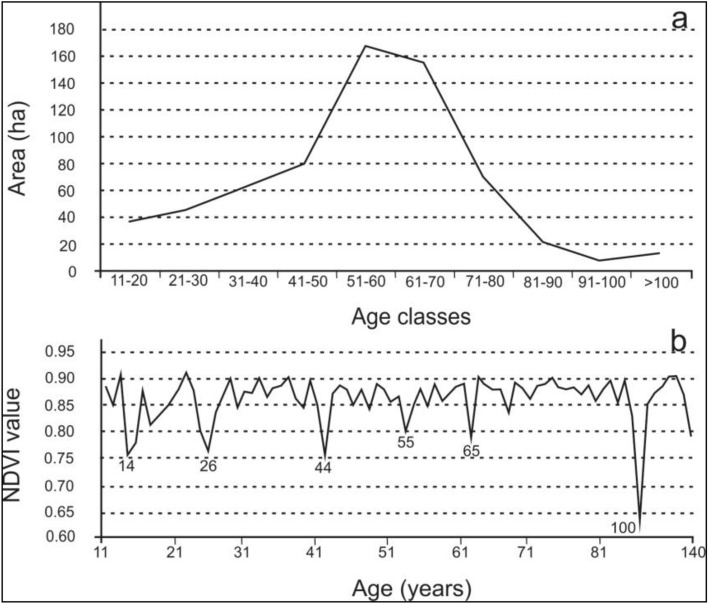
Area distribution of sycamore stands in age classes (**a**) and differentiation of NDVI depending on the age (**b**) of sycamore stands in Poland.

Figures 1 and 2 and were prepared using Microsoft Excel, part of Microsoft Office software used by author according to legal license (Office Home and Student 2021, No: 001SE099381X100798, 79G-05418).

**Figure 3:**
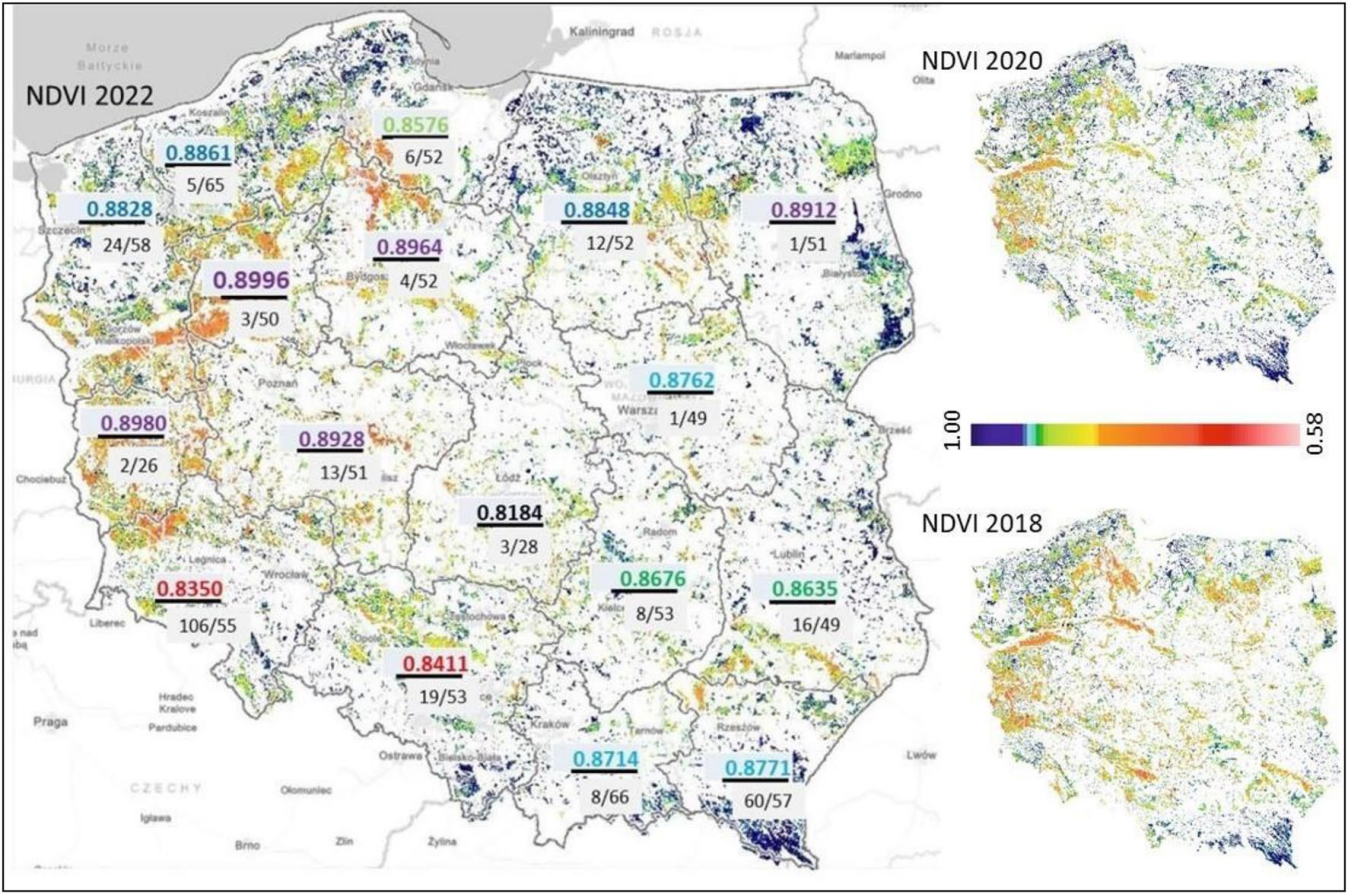
Variation (in the numerator) of mean NDVI values for sycamore, grouped by color into the highest (purple), high (blue), medium (green), low (red), and extremely low (black) values in individual Regional Directorates of the State Forests; in the denominator, before the slash, the number of sycamore stands in a given RDSF, after the slash, the average age in a given RDSF; in the background, an NDVI diversity map covering all stands in Poland as of the first 2 weeks of August 2022, 2020 and 2018, where dark blue represents the highest NDVI values, red the lowest.

Figure 3 is a fully original figure made by the authors. The image of the distribution of stands with a given NDVI value was developed in the QGIS 3.28.3 “Firenze” free available program (https://download.qgis.org/downloads/QGIS-OSGeo4W-3.28.3-1.msi). The values for the Regional Directorates of the State Forests have been overlaid in PowerPoint (part of Microsoft Office software) on this image.

**Figure 4:**
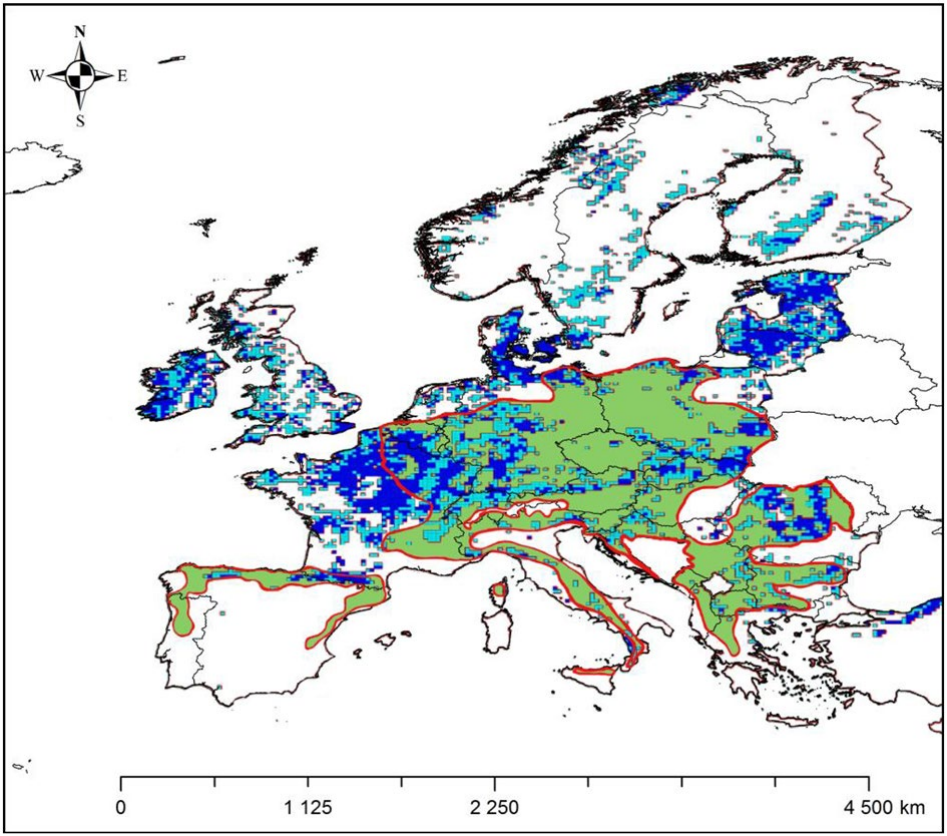
Distribution (in the WGS 1984 coordinate system) of forest areas with NDVI in August 2018 with NDVI ≥ 0.86 (average NDVI value for sycamore stands in Poland); green area and red outline—current natural range of sycamore according to EUFORGEN (https://www.euforgen.org/species/acer-pseudoplatanus/).

Figure 4 is made by the authors in the QGIS 3.28.3 “Firenze” program using NDVI results of our study, overlaid on the natural range of sycamore according to cited source of data:

EUFORGEN (https://www.euforgen.org/species/acer-pseudoplatanus/).

**Figure 5:**
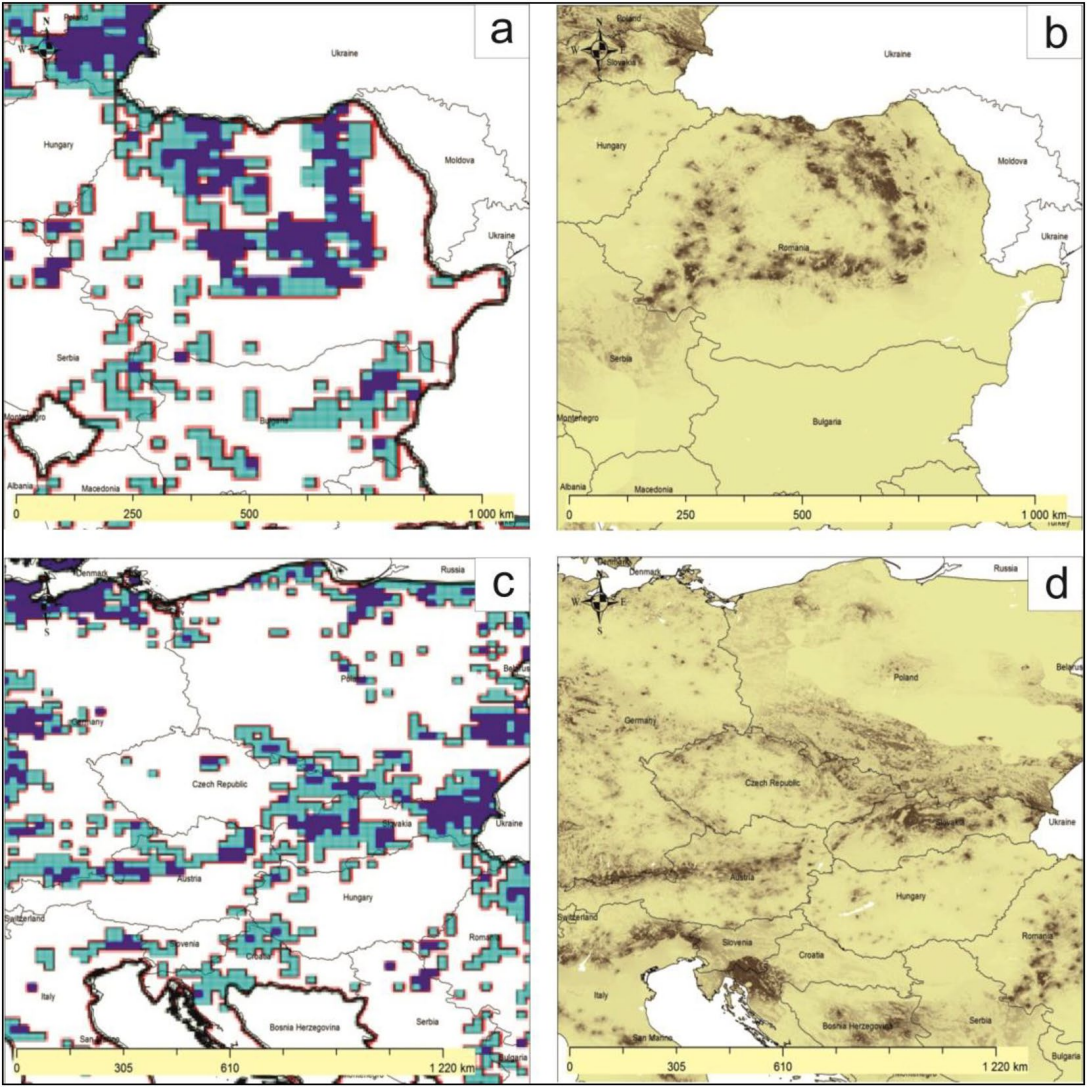
Comparison of tree stands in Romania (**a**) and from Switzerland through Austria to Slovakia and Poland (**c**) with NDVI ≥ 0.86 and estimating the relative probability of presence of *Acer pseudoplatanus* according to de Rigo et al.^27^ (**b**, **d**).

Figure 5a and c are fully original figure made by the authors in the QGIS 3.28.3 “Firenze” program. Figure 5b and d are made by the authors in the QGIS program on the basis of cited source of data [de Rigo, D., Caudullo, G., Houston Durrant, T. & San-Miguel-Ayanz, J., The European Atlas of Forest Tree Species: modelling, data and information on forest tree species. In: San- Miguel-Ayanz, J., de Rigo, D., Caudullo, G., Houston Durrant, T. & Mauri, A. (Eds.), European Atlas of Forest Tree Species. published Off. EU, Luxembourg, pp. e01aa69+. https://w3id.org/mtv/FISE-Comm/v01/e01aa69 (2016)].

## Results

In total, 291 stands were found in Poland, in which the share of sycamore is at least 80%. Their total area is 668.31 ha (Table 1). The largest share of sycamore was noted in eutrophic mesic habitats, both in mountain, upland and lowland forests. The second group in terms of area share are mesotrophic mesic habitats, also representing groups of mountain, upland, and lowland forests. The third place is occupied by a group of eutrophic moist forests, also in 3 altitude varieties (mountain, upland, and lowland). Apart from these three main groups, the presence of sycamore forests along watercourses (alluvial forests), both in the mountains, uplands and lowlands, and occasionally also in habitats considered swampy or oligotrophic.

**Table 1:** Share of sycamore in forest habitat site types.

Forest type	Acronym	Area (ha)	No. of stands
Mountains eutrophic mesic forest	MEMF	228.02	71
Upland eutrophic mesic forest	UEMF	140.94	67
Lowland eutrophic mesic forest	LEMF	113.19	56
Upland mesotrophic mesic forest	UMMF	42.36	18
Mountains mesotrophic mesic forest	MMMF	41.74	24
Lowland mesotrophic mesic forest	LMMF	35.45	18
Lowland eutrophic humid forest	LEHF	21.28	12
Mountains eutrophic humid forest	MEHF	14.34	6
Upland eutrophic humid forest	UEHF	6.70	3
Alluvial upland eutrophic forest	AUEF	5.49	4
Lowland oligotrophic mesic forest	LOMF	5.20	3
Lowland mesotrophic humid forest	LMHF	3.95	3
Mountains oligotrophic mesic forest	MOMF	3.39	2
Alluvial mountain eutrophic forest	AMEF	2.13	1
Lowland mesotrophic swampy forest	LMSF	1.61	1
Lowland alluvial (ash-alder) forest	LAF	1.51	1
Lowland mesic poor pine forest	LMPPF	1.01	1
Total		668.31	291

The calculated average NDVI value for sycamore forests was 0.859, which places this species in 14th place among all (45) species forming forest stands in Poland (Table 2) and 7th among native species in Poland. It is worth noting that the top 5 places on the list are occupied by species alien to the flora of Poland. This can be explained by the fact that these species are usually planted in more fertile habitats, which is reflected in higher NDVI values. This may be supported by the comparison of average NDVI values for sycamore in forest site types (Table 3), where out of seven sites with an NDVI value above the average, five are eutrophic mesic or moist forests. At the same time, the last three places are occupied by the poorest habitat types, with the last one (lowland mesic poor pine forest) being generally considered unsuitable for deciduous tree species in Poland. It can therefore be assumed that reflects well the trophic conditions of sycamore (and not only sycamore) habitats.

**Table 2:**
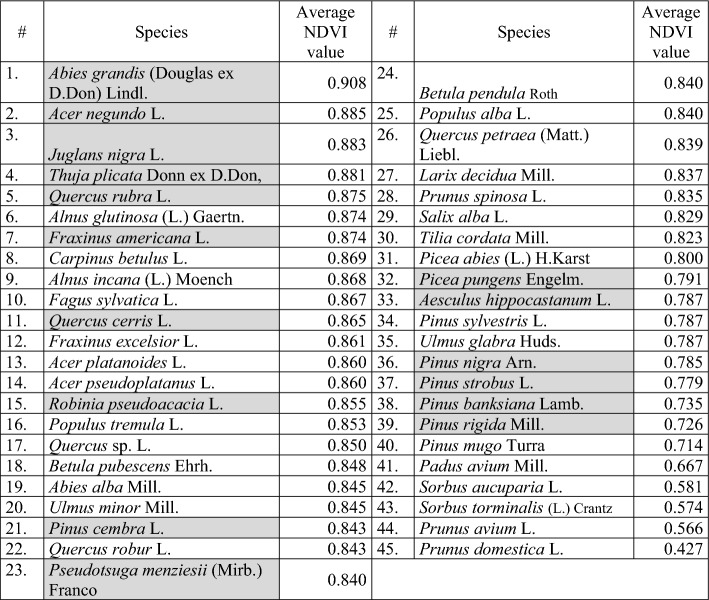
Average NDVI values for the main species in forests in Poland.

Geographically alien species are marked with a gray background.

**Table 3:** Average NDVI value for sycamore stands in forest site types.

#	TSL	NDVI average
**1.**	**Lowland eutrophic mesic forest**	**0.886**
**2.**	**Lowland mesotrophic humid forest**	**0.885**
**3.**	**Lowland eutrophic humid forest**	**0.884**
**4.**	**mountains eutrophic humid forest**	**0.883**
**5.**	**Lowland mesotrophic mesic forest**	**0.875**
**6.**	**Upland eutrophic humid forest**	**0.867**
**7.**	**Upland eutrophic mesic forest**	**0.864**
8.	Lowland alluvial (ash-alder) forest	0.857
9.	Upland mesotrophic mesic forest	0.853
10.	Alluvial upland eutrophic forest	0.849
11.	Mountains mesotrophic mesic forest	0.839
12.	Lowland mesotrophic swampy forest	0.838
13.	Mountains eutrophic mesic forest	0.838
14.	Alluvial mountain eutrophic forest	0.835
15.	Mountains oligotrophic mesic forest	0.815
16.	Lowland oligotrophic mesic forest	0.792
17.	Lowland mesic poor pine forest	0.769
Mean		0.8596

Forest site types with value above the average for all sycamore stands are marked in bold.

Taking into account the data from Table 2, sycamore could be placed in the trophic-humidity series, among the species native to Poland, after *Alnus glutionosa*-*Carpinus betulus-Alnus incana*-*Fagus sylvatica*-*Fraxinus excelsior*-*Acer platanoides*, with sycamore almost equal to NDVI values of common maple.

Sycamore forests in the age range of 50–70 years have the largest area share (Fig. 2a) and this is the range that includes the average age of all forest stands in Poland (60 years)^29^.

The NDVI value was also compared with the age of sycamore stands in Poland (Fig. 2b). It cannot be said on its basis that the NDVI value changes with age, although the graph shows more visible decreases at the age of 14, 26, 44, 55, 65, and finally at the age of 100.

Figure 3 shows a graphical variation of the average NDVI values calculated for the first two weeks of August 2022 for stands representing all species in Poland. The figure shows the boundaries of the main units of the territorial division of the State Forests (Regional Directorates of the State Forests), for which the average NDVI value for sycamore stands in a given RDSF was calculated. The figure also shows the number of sycamore stands with a share of 80–100%, as well as the average age of sycamore stands in a given RDSF. August was chosen due to the results of the study by Młynarczyk et al.^13^ indicating that the optimal period for analyzing the relationship between NDVI and vegetation in Poland is July–August.

Based on Fig. 3, it can be concluded that the average NDVI value for sycamore stands, similarly to the commentary to Fig. 2, does not depend on age, since RDSF Zielona Góra (0.8980) has one of the highest NDVI and the lowest average age (26 years), and Łódź, with a similar average age (28 years) the lowest NDVI (0.8184). In addition, NDVI images for all forest stands in Poland for 2018, 2020 and 2022 show clear differences in the condition of forest stands between the northern, central and southern parts of the country, depending on the distribution of precipitation (higher sum in the north and south compared to the central part).

In order to validate the collected data, an analysis was carried out based on five algorithms (LDA, QDA, SVC, Random Forest and MLP), testing four variants (a, b, c, d) of the relationship between forest habitat type, NDVI, sum of rainfalls, air temperature and soil moisture in each of the algorithms, described below:

- a—classified into 3 habitat classes varied on: lowland (L—95 cases), upland (U—92) and mountain (M—104);
- b – classified into 3 habitat classes varied on: oligotrophic (O—6), mesotrophic (M—63) and eutrophic (E—222);
- c—classified into 4 habitat classes varied on: mesic (263), moisty (22), alluvial (5) and swampy (1);
- d—classified into 8 habitat classes varied on: oligotrophic lowland (OL—2), mesotrophic lowland (ML—21), eutrophic lowland (EL—69); mesotrophic upland (MU—18), eutrophic upland (EU—70); oligotrophic mountain (OM—5), mesotrophic mountain (MM—24), eutrophic mountain (EM—82).

The results are presented in Table 4.

**Table 4:** Data validation results based on five algorithms testing four variants of the relationship between: forest habitat type, NDVI, sum of rainfalls, air temperature and soil moisture.

Algorithm	Test variant			
	a	b	c	d
LDA	0.76	0.88	0.88	0.56
QDA	0.78	0.80	–	0.66
SVC	0.78	0.88	0.88	0.47
RF	0.78	0.88	0.88	0.68
MLP	0.71	0.78	0.93	0.49

The most non-linear model is the MLP model, which clearly distinguishes class differentiation, emphasizing in particular the relationship between the average NDVI value, the sum of precipitation, the average air temperature and the average soil moisture and forest habitat types, depending on the degree of habitat moisture (variant c).

Obtaining a positive validation result, the average NDVI value given in Table 2. (0.860) referred to the European range of sycamore. Figure 4 shows the NDVI values from 0.86 upwards for stands throughout Europe as of August 2018. The value of 0.86 was taken on the basis of the average value for sycamore stands in Poland (0.859), stating that what is above this value is a measure of more favorable habitats for sycamore (Supplementary file).

## Discussion

The results indicating tree stands with NDVI in Europe with a value exceeding the average NDVI value for sycamore in Poland show convergence with maps of the relative probability of presence of *Acer pseudoplatanus* and the maximum habitat suitability for this species^27^. This can be seen, for example, both in Romania and in the belt from southern Germany and Switzerland to southern Poland (Fig. 5).

NDVI ≥ 0.86 in the British Isles, Denmark, Lithuania, Latvia, and Estonia, suggesting suitable conditions for sycamore in these parts of Europe, is considered particularly important. This would be consistent with information about the invasive role of sycamore in Great Britain^6^ or Lithuania^30^. It can be assumed that the species becomes invasive if the habitat conditions are favorable. The mere predisposition to invasiveness is not enough to spread the species. It should also be related to the paper of Tillisch^9^, who suggested that the natural range of sycamore could include Denmark, as well as part of Great Britain, which may be confirmed by the presented research results indicating suitable habitat conditions expressed as NDVI. Tillisch also drew attention to the important issue of human impact on the occurrence of sycamore. This thread is particularly well visible in Poland, although poorly documented in scientific literature. The natural share of sycamore in Poland is limited to mountain forest plant communities, although the natural occurrence of sycamore in mountainous regions of the country may result not only from favorable climatic conditions there, but also from places more difficult to management by humans, which allowed a more natural composition of forest communities to be preserved. However, two-thirds of Poland’s area consist of easily accessible lowland areas, where forests have been left mainly in less fertile areas, and fertile soils have been taken over by agriculture. On the other hand, in the forests of lowland areas, forests are managed and the key species are pine, oak, and beech. However, sycamore is not mentioned in any of the documents regulating forest management in the lowland part of Poland as the main species. As a result, the lack of a formal base for planting sycamore stands means that sycamore was not only not cultivated, but was often eliminated as a species threatening the main species. On the other hand, sycamore was gladly planted as a roadside tree, creating alleys, some of which continue to have a high natural and cultural importance. In many cases, such sycamore alleys, growing along roads running near forest areas, gave rise to the young generation of sycamore trees, which entered under the canopy of the dominant pine in Poland, currently creating two-layered stands, with pine in the upper layer and sycamore in the lower one. Once the pine is removed, sycamore becomes the main species. This would be consistent with the distribution of sycamore stand areas by age class shown in Fig. 2a. The impact of forest management on sycamore stands would also be evident in the data shown in Fig. 2b, where roughly a 10-year cycle between 14 and 65 years of age may be related with periodically performed in Polish forestry treatments of tree stand thinning, with the achievement of a minimum at the age considered to be the age of felling (100 years). Therefore, in assessing the range of sycamore—not only in Poland, but also in other countries—the impact of human activity should be considered significant. Moreover, this impact may be related to both the promotion of sycamore and its elimination. It can be promoted due to its valuable wood, strong root system, resistance to winds, resistance to environmental pollution, low costs of renewal, since it regenerates naturally, and it can be eliminated by, among others, due to the criteria applicable in the assessment of natural habitats in the European network of protected areas called Natura 2000, where in Poland, apart from one type of natural habitat (9180), it is not considered as a desirable species. Hence the opinion about its invasiveness, if it enters uncontrolled forest plant communities forming natural habitats, a situation that mainly concerns oak-hornbeam forests in Poland. On the other hand, in managed forests, the increasing acceptance of sycamore by foresters is observed. Therefore, in assessing changes in the area of this species, it is difficult to separate the impact of climate change due to human activity. Additionally, many European tree species still have unfilled niches because they did not complete their migration after the ice sheet had receded. Sycamore maple is well adapted to the current climatic conditions of central Europe (Straigyte and Baliuckas^8^) but Svenning and Skov^31^ suggest that most of European tree species fill less than 50% of their potential climatic range. Thus, it cannot be ruled out that the expansion in the Baltic States is filling niches that were already colonizable before climate change. Our research confirms earlier predictions that climate changes forecasted for Central Europe in the coming decades may prove beneficial for Acer pseudoplatanus. Unlike many other native species, this species will be gaining potential climatic niches^32^. However, a more precise assessment of the extent of range changes, as indicated by recent studies, requires a broader sampling of climate data from entire geographical ranges and consideration of the latest climate change scenarios^33,34^.

Based on the results of the presented research and comparing it with the coverage maps provided by Szafer^23^ and Boratyński^24^ (Fig. 1), it can be confirmed that in central Poland sycamore finds unfavorable conditions. Contrary to the research results of these authors, it can be assumed that at least currently sycamore finds potentially better conditions in northern Poland than in south-western Poland. This may be partly due to climate change. This could also confirm the predictions of Mauri et al.^20^ indicating the spread of sycamore towards the north of Europe. The presented research results would especially confirm the forecast of Mauri et al.^20^ on the spread of sycamore towards Lithuania, Latvia, and Estonia (Fig. 4). They also do not contradict the lack of Mauri et al.^20^ of sycamore in the British Isles and the results of Morecroft et al.^6^ who suggest that sycamore may decline under climate change if summer droughts, since Morecroft et al.^6^ investigated sycamore sites in central part of England (Wytham Woods), and the results of our research suggest that sycamore finds more suitable conditions in the western part of the British Isles, in particular in Ireland. According to our research in the central part of England, NDVI is less favorable for sycamore.

A side effect of our research is drawing attention to the ranking of average NDVI values for all species forming forest stands in Poland, in which the first 5 places are occupied by species alien to the flora of Poland. It can be assumed that this is due to the introduction of these species to more fertile habitats, which may result in higher biomass and better condition. In this ranking, the first place among Polish natural species is occupied by *Alnus glutinosa*, a species of fertile, moist habitats, which could confirm the results of Młynarczyk et al.^13^ that NDVI also reflects variations in moisture conditions. This seems logical considering that water plays a key role in plant development. In this ranking, sycamore is on the 7th place among species native to the flora of Poland, with the top 7 species being of fertile deciduous forests, mainly oak-hornbeam forests (Table 2—*Carpinus betulus*) and beech forests (Table 2—*Fagus sylvatica*), as well as fertile riverside lowland forests (Table 2—*Fraxinus excelsior*) or mountain forests (Table 2—*Alnus incana*). It can therefore be assumed that NDVI is also a reflection of the trophicity of forest communities, which was already suggested by the results of Młynarczyk et al.^13^. This thesis is also confirmed by the research results listed in Table 3, where forest habitats classified in Poland as the poorest, more typical for pine than deciduous species, occupy the last 3 places in the table presenting the average NDVI values for sycamore stands in the system of forest site types.

The result of the study that should be discussed is the practically constant average NDVI value with changing age, excepting anomalies which may be related to periodic thinning operations carried out in managed forests (Fig. 2b). For many years NDVI has been used for biomass studies^35–37^, especially in cereal research^38^, but measuring of fields with of single-species, annual plants is much simpler than estimating the biomass of a multi-species forest with a complex structure. It is assumed that stand biomass increases with age^39^, but aboveground biomass of some European tree species is poorly characterized^40,41^. Sycamore is also one of these species. As was mentioned in “Introduction” section, the relationship between NDVI and vegetation is very complex. This is illustrated by non-linear biomass of tested sycamore stands at different ages. As reported by Repo et al.^42^ biomass changes in forests of different ages by site and soil types, which also applies to the studied stands. Referring to the methodological assumption, according to which stands with a share of sycamore at least 80% were selected for the study, the relatively even NDVI of the examined stands can be explained by the fact that the share of sycamore that was methodically assumed takes place where habitats are more fertile. In other words, when selecting potential sycamore habitats, one should look for places with higher NDVI values, as shown in Fig. 4.

## Conclusions

The results of the research allow the following conclusions to be drawn:

1. Among the analyzed types of forest habitats (eutrophic, mesotrophic, oligotrophic), differentiated into mountain, upland, and lowland forms, sycamore stands achieve the highest NDVI value in lowland eutrophic mesic forest (0.886).
2. The average NDVI values of sycamore stands are among the highest among all stands in Poland (0.8596) and are almost equal to the values for *Acer platanoides*.
3. Among the stands where the share of sycamore is at least 80%, sycamore stands aged 50–70 years predominate in Poland and this is the range that includes the average age of all forest stands in Poland (60 years). These sycamore stands show a cyclical decrease in NDVI values and a return to average values, which may be related to periodic thinning operations carried out in managed forests.
4. Apart from the southern, mountainous part of Poland, where the share of sycamore stands is the largest, the highest average NDVI values measured for all tree species stands are found in the northern part of Poland, which has so far been considered less favorable for sycamore. This may suggest the potential for an increase in the share of sycamore towards the north.
5. The results of the study may confirm the forecasts given in the literature regarding the spread of sycamore towards Lithuania, Latvia. and Estonia.
6. The results also suggest Denmark and the western part of the British Isles as potentially favorable habitats for sycamore.

### Supplementary Information

Supplementary Information 1.

Supplementary Information 2.

Supplementary Information 3.

Supplementary Information 4.

## Data Availability

The datasets used and/or analyzed during the current study available from the corresponding author on reasonable request.
